# Exploring the Genotype–Phenotype Correlations in a Child with Inherited Seizure and Thrombocytopenia by Digenic Network Analysis

**DOI:** 10.3390/genes15081004

**Published:** 2024-07-31

**Authors:** Shuanglong Lu, Zhixiao Niu, Xiaohong Qiao

**Affiliations:** Department of Pediatrics, Tongji Hospital, Tongji University School of Medicine, 389 Xincun Road, Shanghai 200065, China; 1400456@tongji.edu.cn (S.L.); 2131143@tongji.edu.cn (Z.N.)

**Keywords:** digenic network analysis, seizure, thrombocytopenia

## Abstract

Understanding the correlation between genotype and phenotype remains challenging for modern genetics. Digenic network analysis may provide useful models for understanding complex phenotypes that traditional Mendelian monogenic models cannot explain. Clinical data, whole exome sequencing data, in silico, and machine learning analysis were combined to construct a digenic network that may help unveil the complex genotype–phenotype correlations in a child presenting with inherited seizures and thrombocytopenia. The proband inherited a maternal heterozygous missense variant in *SCN1A* (NM_001165963.4:c.2722G>A) and a paternal heterozygous missense variant in *MYH9* (NM_002473.6:c.3323A>C). In silico analysis showed that these two variants may be pathogenic for inherited seizures and thrombocytopenia in the proband. Moreover, focusing on 230 epilepsy-associated genes and 35 thrombopoiesis genes, variant call format data of the proband were analyzed using machine learning tools (VarCoPP 2.0) and Digenic Effect predictor. A digenic network was constructed, and *SCN1A* and *MYH9* were found to be core genes in the network. Further analysis showed that *MYH9* might be a modifier of *SCN1A*, and the variant in *MYH9* might not only influence the severity of *SCN1A*-related seizure but also lead to thrombocytopenia in the bone marrow. In addition, another eight variants might also be co-factors that account for the proband’s complex phenotypes. Our data show that as a supplement to the traditional Mendelian monogenic model, digenic network analysis may provide reasonable models for the explanation of complex genotype–phenotype correlations.

## 1. Introduction

A traditional Mendelian monogenic model often correlates the genotype with phenotype at a “single-disorder” paradigm, which means a single genetic condition accounts for all the clinical features of patients [1]. However, with the expanding use of next-generation sequencing (NGS), researchers realized that the real genetic mechanism of some diseases may be more complex than we thought. When phenotypes in a family pedigree do not fit a monogenic segregation pattern, exhibiting non-Mendelian inheritance, incomplete penetrance, or phenotypic variability, it is difficult for us to identify genes implicated in diseases. More genetic models are needed to clarify the complex genotype–phenotype correlations in such cases.

When two variants are implicated to describe the phenotypes of patients, there might be three different types of variant combinations. The first one is true digenic: The simultaneous presence of two pathogenic variants is required to produce the phenotypes of the disease; a variant at one of these loci alone does not result in the phenotype. The second one is monogenic plus modifier: a variant on the major gene induces a disease phenotype while another variant in the modifier gene may relieve or aggravate the phenotype. The third one is dual molecular: two variants at different loci are responsible for either distinct or overlapping phenotypes for two different diseases [2,3,4,5,6,7]. Digenic network analysis can predict these candidate disease-causing variant combinations and distinguish digenic inheritance (variants at two different loci result in a simple or syndromic genetic disease) from dual molecular diagnosis (variants at two different loci result in two monogenic disorders), hence revealing us how different genetic conditions and molecular mechanisms could be combined in a single patient. Furthermore, it may also help us find more genetic targets for the treatment of some genetic diseases [8,9,10].

*SCN1A*, located at 2q24.3, encodes the α 1 subunit of the sodium channel and is one of the most important epilepsy genes in humans. Different pathogenic variants of *SCN1A* can have different effects on sodium channel function, which may lead to a wide range of phenotypes with different disease severities, including Dravet syndrome (DS, OMIM #607208), developmental and epileptic encephalopathy 6B (DEE, OMIM #619317), generalized epilepsy with febrile seizures plus type 2 (GEFS+, OMIM #604403), and familial hemiplegic migraine (FHM, OMIM #609634) [11]. However, the phenotypic variability in families with the same variant remains unexplained by a traditional Mendelian monogenic model [12,13].

*MYH9*, located at 22q12.3, encodes the heavy chain of non-muscle myosin IIA, an actin-binding molecular motor that is essential for development. Pathogenic variants of *MYH9* lead to an array of autosomal dominant disorders, including macrothrombocytopenia and granulocyte inclusions with or without nephritis or sensorineural hearing loss (OMIM #155100) and deafness, autosomal dominant 17 (OMIM #603622) [14]. The phenotypes of *MYH9*-related disease (*MYH9*-RD) are also variable among patients in the same family, with different severity and different organ involvement [15,16].

Here, we report a child with pathogenic missense variants in *SCN1A* and *MYH9* simultaneously, presenting with phenotypes of recurrent seizures and thrombocytopenia. By combining clinical data, whole exome sequencing data, in silico, and machine learning analysis, a digenic network map was constructed, which may provide a reasonable explanation for the complex genotype–phenotype correlations in this patient and his family.

## 2. Materials and Methods 

### 2.1. Patient

Blood samples and clinical data were collected from the proband, his parents, his maternal aunt, and his cousin. Written informed consent was obtained from the proband’s parents. 

### 2.2. Whole Exome Sequencing (WES) and Sanger Validation

DNA was extracted from the blood samples using a Genomic DNA Extraction Kit (MyGenostics Kangwei century, Beijing, China). Extracted DNA was fragmented by sonication to an average size of 180 bp using a Bioruptor^®^ (Diagenode, Seraing, Belgium) and then used as a template for library preparation. A P039-Exome probe was hybridized with a DNA library to capture the target gene by MyGenostics Technology Co., Ltd., Beijing, China. The enrichment libraries were sequenced on an Illumina HiSeq X ten sequencer for paired read 150 bp. The resulting reads were quality-controlled, trimmed, and mapped against the human reference genome hg19. Variants were further annotated by ANNOVAR, and some variants identified by WES were amplified from genomic DNA by polymerase chain reaction (PCR) and then sequenced by automatic genetic analyzer ABI 3730xl (Applied Biosystems, Foster City, CA, USA).

### 2.3. Variant Analysis and Interpretation

Variants were annotated through multiple databases, such as the 1000 Genomes, ESP6500, dbSNP, EXAC, in-house (MyGenostics), and HGMD, predicted by MutationTaster 2021 (https://www.genecascade.org/MutationTaster2021 (accessed on 1th June 2024)), and classified according to the Association for Molecular Pathology/American College of Medical Genetics and Genomics (ACMG) guidelines [17]. Protein mutation analysis, including the protein residue conservation of mutated positions across different species and the variant’s possible impact on the protein’s function, was carried out by the VarSite web server (https://www.ebi.ac.uk/thornton-srv/databases/VarSite (accessed on 1 June 2024)) [18]. Two core variants were submitted to the Leiden Open Variation Database (LOVD) with individual ID 00,448,671 (https://databases.lovd.nl/shared/individuals/00448671 (accessed on 1 June 2024)).

### 2.4. Digenic Network Analysis 

The variant call format (VCF) data annotated by ANNOVAR were submitted to the bioinformatics platform ORVAL (Oligogenic Resource for Variant AnaLysis, https://orval.ibsquare.be (accessed on 1 June 2024)) [19] for the exploration of disease-causing variant combinations. The genome version was set as GRCh37/hg19, and the patient information was set as male. Variant filtering was set at a Minor Allele Frequency (MAF) lower than or equal to 3.5% in protein-coding genes to remove intergenic and intronic/synonymous variants. Gene sets containing 230 epilepsy-associated genes [20] and 35 thrombopoiesis genes [21] reported so far were used as gene-filtering panels to ensure that the analysis was consistent with the patient’s phenotype. 

The machine learning tools Variant Combination Pathogenicity Predictor (VarCoPP 2.0) [22] and the Digenic Effect predictor [23] based on the Digenic Diseases Database (DIDA) [24] and Oligogenic Diseases Database (OLIDA) [25] were used to predict bi-locus variant combinations pathogenicity and digenic combination effects. Gene pair pathogenicity score greater than 0.891 was set as the network filter, and the final digenic network was constructed. VarCoPP 2.0 is a balanced Random Forest (RF) predictor that consists of 400 decision trees. It has been trained on the pathogenic variant combinations present in the OLIDA database against a large subset of variant data derived from control individuals of the 1000 Genomes Project (1KGP). Based on the variant pairs’ biological features, including Combined Annotation Dependent Depletion (CADD) raw score, gene haploinsufficiency prediction, inheritance-specific pathogenicity prediction, selective pressure, biological distance, biological process similarity, and distance in an in-house developed Knowledge Graph, VarCoPP 2.0 can predict the pathogenicity score (value between 0 and 1) of any bi-locus variant combination. If the gene pair pathogenicity score was greater than 0.891 (genome version hg19), it had a 99.9% probability of being a true positive disease-causing variant combination. The Digenic Effect predictor is a classification Random Forest (RF) predictor trained on 240 pathogenic variant combinations. It uses the variant pairs’ biological features, including the CADD raw score, gene recessiveness probability, essential in mice, and the same pathway to predict digenic combination effects, including true digenic combinations, monogenic plus modifier combinations, and dual-molecular diagnosis combinations.

### 2.5. Protein–Protein Interactions and Pathways Analysis

Those 10 genes passing the >0.891 gene pair pathogenicity score filter were employed for the construction of the digenic network and subsequently uploaded to the STRING database (version 12.0; https://string-db.org (accessed on 1 June 2024)) [26] to explore Protein–Protein Interactions, and the interaction type was set as the full STRING network, with the minimum required interaction score set as medium confidence (0.40). These 10 genes were also uploaded to the Reactome Pathway Knowledgebase 2024 (https://reactome.org (accessed on 1 June 2024)) [27] to explore the biological pathways of these proteins, and the main and common biological pathways of key genes were mapped.

### 2.6. Gene Expression and Co-Expression Analysis

To illustrate the expression and co-expression profiles of 10 genes passing the >0.891 gene pair pathogenicity score filter in our digenic network, the transcript expression levels of them in different tissues, based on transcriptomic data from the Human Protein Atlas (HPA) RNA-seq data and Genotype-Tissue Expression (GTEx) project data [28] were downloaded from the HPA database (https://www.proteinatlas.org (accessed on 1 June 2024)). The consensus-normalized expression (nTPM) values of the genes in the cerebral cortex and bone marrow were mapped. 

GEPIA2 [29], a web server for large-scale expression profiling and interactive analysis, was used for the co-expression analysis. Pearson correlations of these 10 gene expressions in the human brain of the GTEx database were calculated, and a co-expression matrix was constructed.

## 3. Results 

### 3.1. Clinical Observations

**Figure 1:**
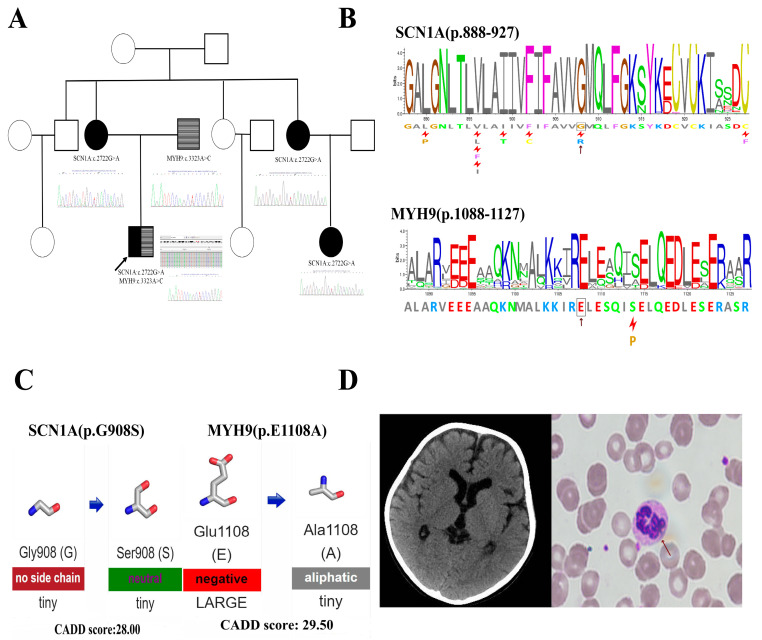
Genetical analysis and clinical data of the patient. (**A**) Pedigree diagram of patients. The black arrow indicates the proband, the genotypes, and the Sanger sequencing results were placed below or beside each family member; the black filling represents seizure phenotype, and the horizontal stripe filling represents thrombocytopenia phenotype. (**B**) Forty residue sequence logos centered on Gly908 of SCN1A and Glu1108 of MYH9. Residue conservations at each position from multiple alignments of homologous proteins were depicted; the taller the letter, the more commonly it is found at that position, and residues marked by the red lightning bolts are known pathogenic variants at that position. (**C**) The property changes of the sidechain for SCN1A (p.G908S) AND MYH9 (p.E1108A). The hydrophobicity, the size of the sidechain, and the Combined Annotation Dependent Depletion (CADD) scores of variants were depicted. (**D**) Head computed tomography (CT) scan image of the patient and the Romanowsky–Giemsa staining of the patient’s peripheral blood smear. The red arrow indicates Döhle body-like cytoplasmic inclusion bodies in the neutrophils.

A 4-year-old boy was referred to our department with a sudden, unexpected onset of fever-associated repeated seizures, without any previous history of seizures, together with a blood finding of thrombocytopenia. Recurrent seizures with fever were observed and manifested as generalized tonic-clonic seizures that lasted up to 10 min. Head computed tomography (CT) showed only a slightly widened supratentorial brain ventricle and extracerebral space (Figure 1D). Electroencephalography (EEG) and cerebrospinal fluid examination results were normal, and the antiepileptic drug levetiracetam was used to control the seizures.

The initial leukocyte count was low at 2.9 × 10^9^/L, including 60.9% lymphocytes and 31.1% neutrophils. Hemoglobin (Hb) was low at 8.5 gm/dL, and his platelet count was very low at 36 × 10^9^/L, indicating thrombocytopenia. Bone marrow smear showed hypocellular marrow with normal erythroid and granulocytic precursors and decreased megakaryocytes. Antiplatelet antibodies and platelet-associated immunoglobulins were not detected. HB and leukocyte count recovered to normal after the fever had settled with antibiotics; however, the platelet count remained low, ranging from 30 to 50 × 10^9^/L. His peripheral blood smear showed Döhle body-like cytoplasmic inclusion bodies in neutrophils (Figure 1D) and some giant platelets, which might be signs of *MYH9*-related disorder. 

No special symptoms or signs, including skeletal abnormalities, microcephaly, mental retardation, or visual or hearing difficulties, were observed. Other laboratory data, including liver and kidney function, electrolytes, coagulation, myocardial enzymes, and echocardiography, were normal. 

Family history (Figure 1A) revealed that the boy’s mother had a single seizure lasting for about 15 s at the age of 28 years old and did not have fever-associated convulsions as a child. The boy’s father had no history of seizures. The boy’s maternal aunt had a single fever-associated convulsion lasting for about 10 s at the age of 5 years old and had no subsequent recurrence. The boy’s maternal cousin had a single fever-associated convulsion lasting for about 10 s at the age of 1 year old and had no subsequent recurrence. All the seizures of the boy, his mother, his maternal aunt, and his cousin were manifested as generalized tonic-clonic, but the boy’s seizures were more frequent and lasted much longer than his mother, maternal aunt, and cousin (Table 1). 

The boy’s mother had no family history of hematological disease. The boy’s father once had mild thrombocytopenia (82 × 10^9^/L) when he had a respiratory infection, and no other special symptoms were found in him. The relatives of the boy’s father had no symptoms of thrombocytopenia.

The boy had been followed up in our department for about 4 years. After the treatment of levetiracetam, the boy’s seizure had been well controlled, only reoccurred once for 5 min in the first year of disease onset, and had no subsequent recurrence. Eltrombopag was given to treat his thrombocytopenia, but his platelet counts were constantly low (ranging from 30 to 80 × 10^9^/L).

### 3.2. WES and Variants Interpretation

WES and Sanger sequencing were performed, and two possible pathogenic variants were identified. The proband had inherited a heterozygous missense variant in *SCN1A* (NM_001165963.4:c.2722G>A) from his mother (Figure 1A). His maternal aunt and cousin also had this variant (Figure 1A). Evidence analysis showed that the variant located in the hotspot region of the *SCN1A* gene (Pathogenic Moderate 1, PM1) was absent from the normal control database (Pathogenic Moderate 2, PM2), changed an amino acid residue where different pathogenic changes had been reported previously (Pathogenic Moderate 5, PM5), and multiple computational evidence supports it as a deleterious variant (Pathogenic supporting 3, PP3). Protein mutation analysis showed that this variant can cause a Glycine-to-Serine residue change at position 908 of the SCN1A protein. Position 908 is highly conserved (Figure 1B, conservation = 1.0, from 200 aligned protein sequences) and was predicted to be very important for the function of the protein. In addition, Glycine is an amino acid with no side chain, and Serine has a neutral side chain; changes from Glycine to Serine may change the flexibility and hydrophobicity of the protein in this position, and the CADD score for this variant is 28, indicating that it might be a deleterious variant (Figure 1C). Finally, the missense variant in *SCN1A* (NM_001165963.4:c.2722G>A) in the proband was classified to be Likely Pathogenic according to the ACMG guideline (PM1 + PM2 + PM5 + PP3).

WES and the Sanger sequencing revealed that the proband inherited another heterozygous missense variant in *MYH9* (NM_002473.6:c.3323A>C) from his father (Figure 1A). Evidence analysis showed this variant had a damaging effect on the gene and protein in vivo (Pathogenic Strong 3, PS3), which was absent from the normal control database (Pathogenic Moderate 2, PM2). Missense variants in *MYH9* have a low rate of benign missense variation. Missense variants are a common mechanism of disease for *MYH9* (Pathogenic supporting 2, PP2), and multiple computational evidence supports it being a deleterious variant (Pathogenic supporting 3, PP3). Protein mutation analysis showed that this variant can cause a Glutamic acid-to-Alanine residue change at position 1108 of the MYH9 protein; position 1108 is also highly conserved (Figure 1B; conservation = 0.8 from 200 aligned protein sequences). In addition, Glutamic acid is a negatively charged hydrophilic amino acid, and Alanine has an aliphatic hydrophobic side chain. Changes from Glutamic to Alanine may change the hydrophobicity of protein in this position. The CADD score for this variant is 29.5, which means it might also be a deleterious variant (Figure 1C). Finally, the missense variant in *MYH9* (NM_002473.6:c.3323A>C) in the proband was classified to be Likely Pathogenic according to the ACMG guideline (PS3 + PM2 + PP2 + PP3).

### 3.3. Digenic Network and Combinations Annotation 

Focused on 230 epilepsy-associated genes [20] and 35 thrombopoiesis genes [21] (Supplementary Table S1), a digenic network for the patient was constructed by machine learning tool VarCoPP 2.0 (Supplementary Figure S1). A total of 3203 variant combinations in 47 genes were found in the network, of which 2903 combinations were predicted to be neutral; a total of 233 combinations were predicted to be candidate disease-causing with low confidence, 45 combinations were predicted to be disease-causing with 99% confidence, and 22 combinations were predicted to be disease-causing with 99.9% confidence. *SCN1A* and *MYH9* were core genes in the network, with the highest network centrality of 29 (Supplementary Table S2). 

To achieve the highest true positive probability results, the gene pair pathogenicity score filter was set at >0.891, and a final digenic network containing 10 genes and disease-causing variant combinations with 99.9% confidence was constructed, including 9 epilepsy-associated genes and 2 thrombopoiesis genes (*DIAPH1* was shared by both phenotypes). 

**Figure 2:**
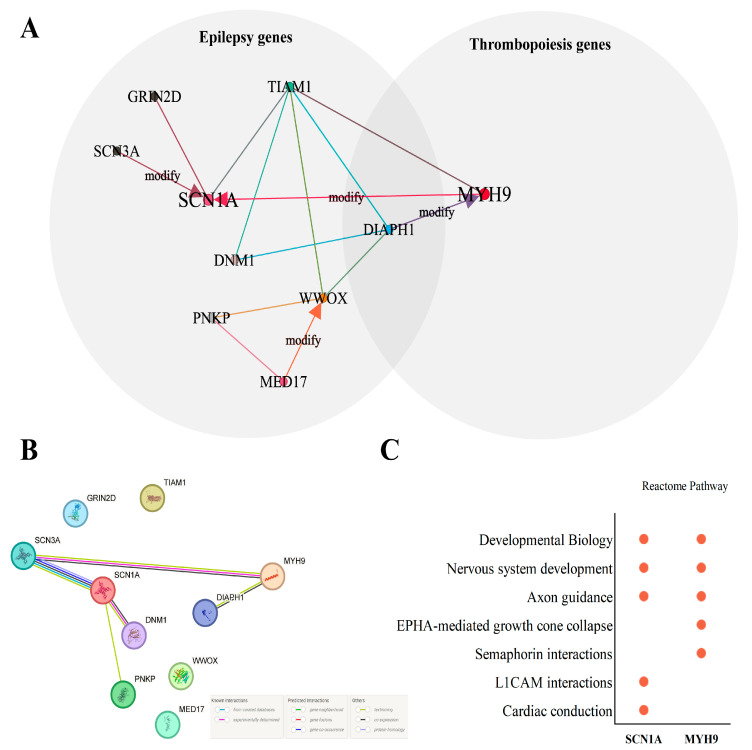
Digenic network of patient and core genes’ relationship analysis. (**A**) Digenic network and Venn map of related genes. Two shadow circles represent types of related genes, lines represent two genes that had digenic combinations with 99.9% true positive probability confidence. The arrow of the line indicates one gene might be a phenotype modifier of another gene. (**B**) Protein–Protein Interactions (PPIs) network of 10 genes in the digenic network. Lines represent the interaction between proteins. (**C**) Reactome pathways of SCN1A and MYH9. The red dot represents the pathways the gene is involved.

Meanwhile, the Digenic Effect predictor revealed that the phenotype of *SCN1A* might be modified by *MYH9* and *SCN3A* and that the phenotype of *MYH9* might be modified by *DIAPH1* in the network (Figure 2A). 

### 3.4. Protein–Protein Interactions and Pathways Analysis of Core Genes

Because there were different relationship types between pairs of genes carrying variants causing digenic disease, including direct interaction, indirect interaction, pathway membership, co-expression, and similar functions [24], to better understand the relationship of gene pairs in the patient’s digenic network, Protein–Protein Interactions (PPIs) network of 10 genes was constructed, which revealed that MYH9 might have indirect interaction with SCN1A through SCN3A. Meanwhile, DIAPH1 was found to have a direct interaction with MYH9, which may help its phenotype modifier function in MYH9 (Figure 2B). 

In addition, Reactome Pathway analysis of the 10 genes showed that SCN1A and MYH9 had common pathways: developmental biology–nervous system development–axon guidance (Supplementary Table S3 and Figure 2C). 

### 3.5. Expression, Co-Expression Profiles of Genes, and Digenic Model for Patient

**Figure 3:**
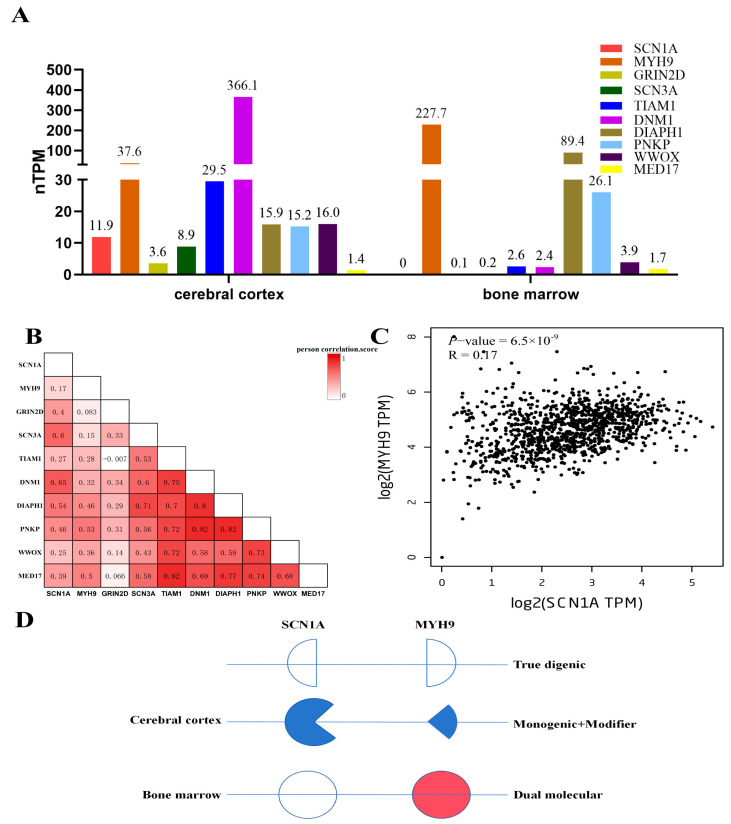
Expression, co-expression profiles of genes, and digenic model for patient. (**A**) Expression profiles of 10 genes in human cerebral cortex and bone marrow. (**B**) Co-expression matrix of 10 genes in the human brain. The numbers inside the box were the value of the Pearson correlation coefficient, and the color depth represents the correlation degree. (**C**) Co-expression map of *SCN1A* and *MYH9* in the human brain. (**D**) Digenic model for explanation of recurrent seizure and thrombocytopenia in the patient. Three types of variant combinations were depicted. Color fillings represent genes that have pathogenic effects, blue represents effects on seizure, and red represents effects on thrombocytopenia.

Because most protein-encoding genes were affected by their expression level, to better understand the relationship of gene pairs, the expression profiles of 10 core genes based on HPA RNA-seq data and the GTEx project data were depicted. Nearly all 10 genes have different degrees of expression in the human cerebral cortex; however, only *MYH9*, *DIAPH1*, and *PNKP* have relatively high expression levels in the human bone marrow (Figure 3A). The co-expression matrix in the human brain shows that the expression of all the genes is positively correlated (Figure 3B), including the gene pair *SCN1A* and *MYH9*, which has a Pearson correlation coefficient of 0.17 (*p* = 6.5 × 10^−9^) (Figure 3C).

Based on the indirect interaction between *MYH9* and *SCN1A*, common pathways, and co-expression, a digenic model was built to explain recurrent seizures and thrombocytopenia in the patient. In the cerebral cortex, the seizure phenotype of *SCN1A* may be modified by *MYH9*, whereas in the bone marrow, *MYH9* may have an independent dual molecular effect, leading to thrombocytopenia (Figure 3D).

## 4. Discussion

The ultimate goal of medical genetics is to reveal the complex genotype–phenotype relationships of genetic disorders. Based on the philosophic tenet of Occam’s razor (the simplest diagnosis is the most likely to be correct) [30], medical geneticists usually use a single genetic diagnosis to explain all the phenotypes of the patient; however, in this “single-disorder” paradigm, the phenotypes do not always fit into the genotype perfectly. With the wide application of WES, the principle of Occam’s razor has been challenged [31,32], and different variant combination models, including true digenic, monogenic plus modifier, and dual molecular models (Figure 3D) have been used to explain complex phenotypes [33,34,35,36]. Here, we reported a patient with complex phenotypes that cannot be diagnosed by the “single-disorder” paradigm. Digenic network analysis was applied, and a reasonable digenic model was established to explain the patient’s seizures and thrombocytopenia.

For the phenotype of recurrent seizure, a maternal heterozygous missense variant in *SCN1A* (NM_001165963.4:c.2722G>A) was found in the patient and was classified to be likely pathogenic according to ACMG guideline, combined with clinical manifestations, the patient and his mother, aunt, and cousin can all be diagnosed as *SCN1A*-related GEFS+ (OMIM #604403). However, the frequency and duration of seizures in the patient were more frequent and much longer than in his mother, aunt, and cousin. Such intra-familial variability in phenotype severity has been observed in some other studies [12,13,37,38] and was explained by mosaicism, variants in regulatory regions, clinical management, or modifier genes. Some genes have been identified as modifiers of *SCN1A*-related seizure before [39,40,41], but all of these were epilepsy-related genes; no *MYH9*-modified seizure has been reported before. 

Our study identified a paternal heterozygous missense variant in *MYH9* (NM_002473.6:c.3323A>C), which was previously considered as a reason for the phenotype of macrothrombocytopenia and granulocyte inclusions, with or without nephritis or sensorineural hearing loss (OMIM #155100). After expression, a PPI network, and pathway analysis, we found that in the cerebral cortex of humans, *MYH9* interacted with *SCN1A* indirectly [42], was co-expressed with *SCN1A*, and had common biological pathways with *SCN1A* for nervous system development [43], especially for modeling synaptic plasticity [44,45]. Hence, we propose that in the cerebral cortex of the patient, the paternal variant in *MYH9* might be a modifier of *SCN1A* and might be an explanation for the seizure phenotype variability observed between the patient and his mother, aunt, and cousin. The *SCN1A*-*MYH9* variant combination in the cerebral cortex fits in the second type of variant combination (monogenic plus modifier).

Meanwhile, for the phenotype of thrombocytopenia, we found that in the bone marrow, the expression of *SCN1A* is very low, indicating that *SCN1A* might have little effect on the patient’s hematological system phenotypes. The thrombocytopenia of the patient can only be explained by the variant of *MYH9*, which means the variant in *SCN1A* accounts for the seizure phenotype, and the variant in *MYH9* leads to thrombocytopenia in the bone marrow of the patient independently. The *SCN1A*-*MYH9* variant combination in bone marrow may also fit in the third type of variant combination (dual molecular).

Additionally, a variant in the epilepsy- and thrombopoiesis-associated gene *DIAPH1* was also identified in our digenic network, but it was not a core gene in our digenic network (gene centrality score was far less than *SCN1A* and *MYH9*, details in Supplementary Table S2) and the patient’s variant in *DIAPH1* was classified as uncertain according to the ACMG guideline. The patient’s symptoms cannot be explained only by the phenotype terms of *DIAPH1* (deafness, autosomal dominant 1, with or without thrombocytopenia, OMIM #124900, seizures, cortical blindness, microcephaly syndrome, OMIM #616632). Hence, we propose it might be only a modifier gene for the patient’s phenotypes in our digenic network, and it may affect *SCN1A*- and *MYH9*-related phenotypes directly or indirectly (Figure 2A). Similarly, another seven genes were identified in our digenic network, which were all classified as uncertain according to the ACMG guidelines; however, as members of the network, they may also be co-factors that account for the proband’s complex phenotypes.

Our study had some limitations. First, we only focused on point variants in the patient. Other genetic conditions, such as copy number variation (CNV) and epigenetic changes, may also be involved in the patient’s complex phenotypes. This will hinder the digenic model’s further application. More database and machine learning tools about CNV and epigenetic changes are needed to improve and validate the digenic model. Second, due to limited conditions, the full pedigree chart of the proband cannot be obtained. This may hinder our interpretation of the digenic network. Third, although the machine learning tool VarCoPP 2.0 predicted the variant combinations in our network and had a 99.9% probability of being a true positive disease-causing variant combination, there were still possibilities of coincidence. The coexistence of two different monogenic disorders cannot be excluded. More experimental studies are needed in the future to validate the disease-causing model of our variant combinations. Similarly, although the Digenic Effect predictor predicted our digenic combination effects into a monogenic plus modifier model, we can only speculate that the modifier effect of *MYH9* may be the reason for the intra-familial variability of phenotype; however, other genetic and environmental factors may also attribute to the severity variability of epilepsy within the family, so more experimental studies are also needed to analyze the functional interaction of *MYH9* and *SCN1A* to support the modifier effect of *MYH9* in our network.

In conclusion, from our digenic network, we proposed that in the cerebral cortex, the variant of *MYH9* might work as a modifier of *SCN1A* and, in this family, led to intra-family seizure phenotype variability, whereas in the bone marrow, the variant of *MYH9* has probably led to thrombocytopenia. Also, another eight genes might be co-factors for our patient’s complex phenotypes. Although our digenic network provided a reasonable model for the explanation of genotype–phenotype correlations in our patient, further evidence is needed to validate it in the future.

## Figures and Tables

**Table 1:** Clinical features of the proband and his affected family members.

Person	Gender	Onset Age (Years)	Type of Seizure	Age Range of Seizures (Years)	Fever/Temp (°C)	Duration of Longest Seizure	Duration of Episode/How Many Seizures Per Episode	Frequency of Episodes	On Treatment at the Time
Patient	M	4	Generalized tonic-clonic	4~5	Yes/39.8	10 min	5 days/20+ times	Twice in one year	Levetiracetam
Mother	F	28	Generalized tonic-clonic	28	Yes/39.5	15 s	Once	–	Supportive treatment
Aunt	F	5	Generalized tonic-clonic	5	Yes/–	10 s	Once	–	Supportive treatment
Cousin	F	1	Generalized tonic-clonic	1	Yes/–	10 s	Once	–	Supportive treatment

## Data Availability

Data for this study are available upon request from the corresponding author.

## Supplementary Materials

The following supporting information can be downloaded at: https://www.mdpi.com/article/10.3390/genes15081004/s1, Figure S1: The total digenic network of patient; Table S1: Gene sets for variants filtering; Table S2: Digenic network genes’ centrality; Table S3: Reactome pathway of 10 genes in the digenic network.
