# Supplementary material for: Exploring the Genotype–Phenotype Correlations in a Child with Inherited Seizure and Thrombocytopenia by Digenic Network Analysis
Source: Genes (Basel). 2024 Jul 31;15(8):1004. doi: 10.3390/genes15081004 (PMC11353731; doi:10.3390/genes15081004)
Supplement: Supplementary file 1 [file genes-15-01004-s001.zip › genes-3095828-supplementary.pdf]

Exploring the Genotype–Phenotype Correlations in a Child with Inherited Seizure and Thrombocytopenia by Digenic Network Analysis

Supplementary Material

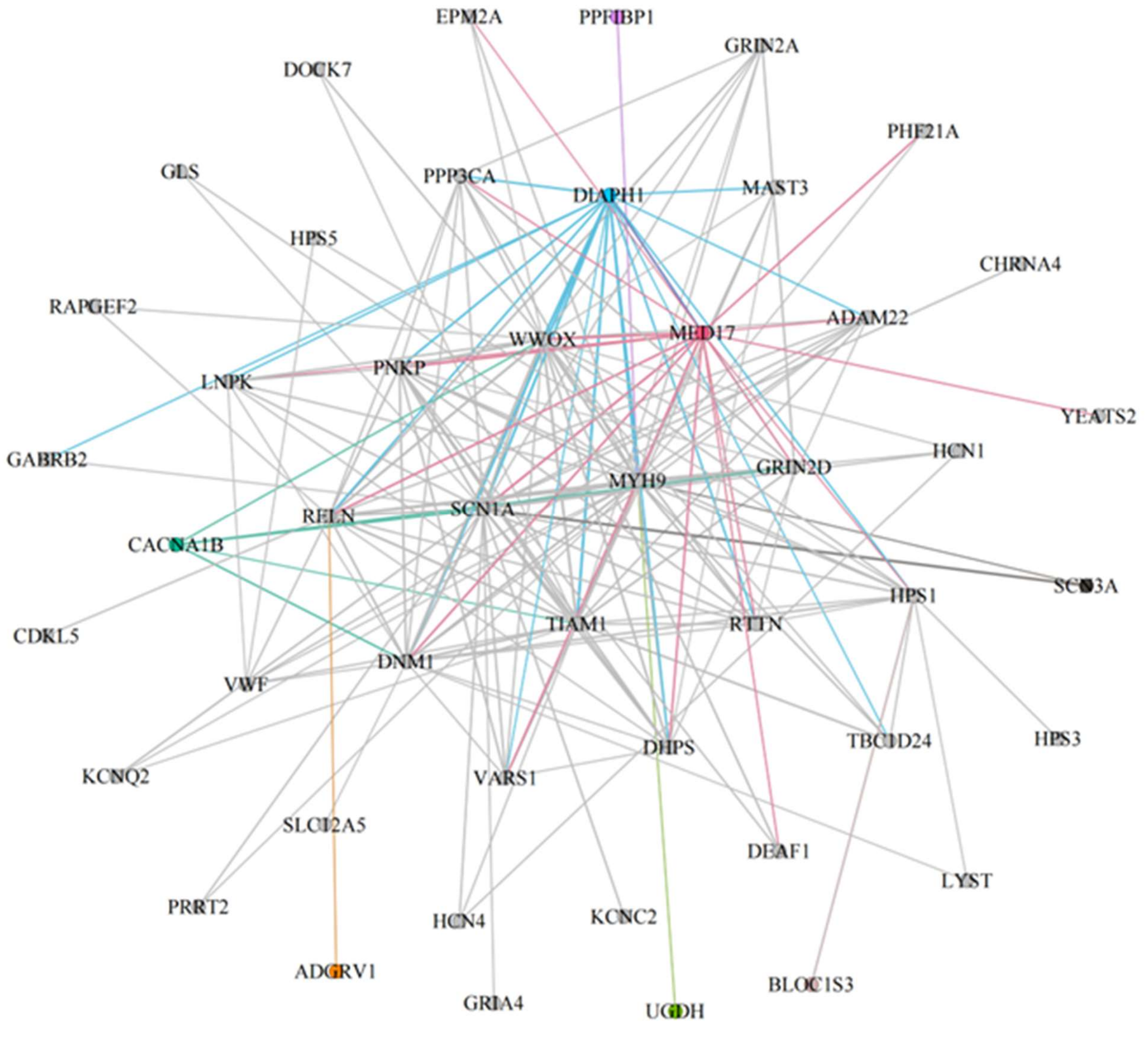

Figure S1. The total digenic network of patient.

Table S1. **Gene sets for variants filtering.**

Gene sets	
**Epilepsy-associated genes**	TNRC6A,RAPGEF2,YEATS2,CNTN2,PRDM8,LMNB2,ADGRV1,KCNT1,SYN1,PLPBP,SETD1A,SAMD12,STARD7,MARCHF6,DEPDC5,NPRL2,NPRL3,SCN3A,LGI1,GRIN2A,CHRNA4,CHRNB2,CHRNA2,CSTB,EPM2A,NHLRC1,KCTD7,SCARB2,GOSR2,KCNC1,SEMA6B,SLC7A6OS,CERS1,ALDH7A1,TBC1D24,HCN2,SCN1A,GABRG2,SCN1B,HCN1,STX1B,SLC6A1,PRRT2,SCN2A,SCN8A,KCNQ2,KCNQ3,GABRA1,GABRB3,RELN,GABRD,CLCN2,SLC2A1,SLC12A5,RORB,KCNMA1,HCN4,MDH1,ARX,FBXO28,GRIN1,SLC38A3,KCNC2,ATP6V0A1,HID1,UFSP2,NAPB,MAST3,PLCB1,ST3GAL3,GNAO1,SZT2,CDKL5,NECAP1,DOCK7,SLC13A5,KCNB1,GRIN2B,WWOX,AARS1,SLC25A22,SIK1,DNM1,KCNA2,EEF1A2,ITPA,ALG13,FRRS1L,ARV1,SLC25A12,STXBP1,SLC1A2,CACNA1A,UBA5,GABRB1,GRIN2D,FGF12,AP3B2,DENND5A,SPTAN1,CAD,MDH2,SYNJ1,HNRNPU,PIGP,YWHAG,KCNT2,NTRK2,GABBR2,CNPY3,ADAM22,CPLX1,RHOBTB2,CYFIP2,PACS2,CUX2,TRAK1,CACNA1E,PHACTR1,GLS,NEUROD2,RNF13,PARS2,ACTL6B,GABRA2,GABRA5,ARHGEF9,PIGB,DMXL2,GOT2,UGP2,UGDH,SMC1A,CDK19,GAD1,PCDH19,FGF13,PPP3CA,GABRB2,ATP6V1A,CHD2,PIGS,NSF,CELF2,ATP1A2,ATP1A3,SLC35A3,CACNA2D2,OXR1,CLN8,ATN1,DHDDS,ARFGEF1,KCNK4,LIAS,CNNM2,ATP1A1,P4HTM,AP2M1,TANC2,PHF21A,OTUD6B,ALG14,PAK1,RORA,PDE2A,SETD1B,NUS1,MED23,YIPF5,IER3IP1,MED17,QARS1,PNKP,RTTN,ADPRS,PTPN23,WASF1,PIGU,BRAT1,EMC10,PIGA,LNPK,NACC1,TRAPPC4,SPATA5,PIGK,SNIP1,DEAF1,CACNA1C,ADARB1,HECW2,GRIK2,NCDN,TIAM1,TRAPPC6B,MTHFS,CPSF3,NARS1,CHKA,VARS1,DLL1,CUL3,NBEA,PIGG,GRIA4,IRF2BPL,CLCN3,EXOC7,TBC1D2B,CACNA1B,DHPS,GRM7,PPFIBP1,WDR45B,NSRP1,CACNA1I,WARS2,DHX16,PIGF,RTN4IP1,TUBGCP2,STRADA,CACNA1D,DIAPH1,NRROS,EXT2,PRMT7,HACE1,ASAH1
**Thrombopoiesis genes**	THPO,MPL,GATA1,RUNX1,FLI1,ETV6,GFI1B,HOXA11,MECOM,ANKRD26,RBM8A,HPS1,A03B1,HPS3,HPS5,HPS6,DTNMP1,BLOC1S3,BLOC1S6,LYST,NBEA,MYH9,WAS,ACTN1,FLNA,DIAPH1,P2Y12,TBXA2R,PTXAS1,ITGA2B,ITGB3,GPIBA,GPIBB,GP9,VWF

Table S2. Digenic network genes' centrality

Ensembl ID	Gene	Centrality	Ensembl ID	Gene	Centrality
ENSG00000144285	SCN1A	29	ENSG00000112425	EPM2A	3
ENSG00000100345	MYH9	29	ENSG00000075043	KCNQ2	3
ENSG00000186153	WWOX	25	ENSG00000138622	HCN4	3
ENSG00000156299	TIAM1	23	ENSG00000145864	GABRB2	2
ENSG00000042429	MED17	20	ENSG00000143669	LYST	2
ENSG00000131504	DIAPH1	19	ENSG00000109756	RAPGEF2	2
ENSG00000106976	DNM1	18	ENSG00000135365	PHF21A	2
ENSG00000189056	RELN	18	ENSG00000153253	SCN3A	2
ENSG00000039650	PNKP	16	ENSG00000115419	GLS	2
ENSG00000107521	HPS1	14	ENSG00000167371	PRRT2	2
ENSG00000095059	DHPS	12	ENSG00000116641	DOCK7	2
ENSG00000105464	GRIN2D	12	ENSG00000110756	HPS5	2
ENSG00000176225	RTTN	11	ENSG00000163872	YEATS2	1
ENSG00000008277	ADAM22	10	ENSG00000164199	ADGRV1	1
ENSG00000138814	PPP3CA	10	ENSG00000008086	CDKL5	1
ENSG00000204394	VARS1	9	ENSG00000189114	BLOC1S3	1
ENSG00000144320	LNPK	9	ENSG00000109814	UGDH	1
ENSG00000110799	VWF	7	ENSG00000110841	PPFIBP1	1
ENSG00000183454	GRIN2A	7	ENSG00000166006	KCNC2	1
ENSG00000162065	TBC1D24	5	ENSG00000152578	GRIA4	1
ENSG00000099308	MAST3	5	ENSG00000101204	CHRNA4	1
ENSG00000148408	CACNA1B	5	ENSG00000163755	HPS3	1
ENSG00000164588	HCN1	4	ENSG00000124140	SLC12A5	1
ENSG00000177030	DEAF1	3			

Table S3. Reactome pathway of 10 genes in the digenic network

Pathway name	Submitted entities found	Pathway name	Submitted entities found
Interaction between L1 and Ankyrins	SCN3A;SCN1A	Translocation of SLC2A4 (GLUT4) to the plasma membrane	MYH9
Phase 0 - rapid depolarisation	SCN3A;SCN1A	Base Excision Repair	PNKP
L1CAM interactions	SCN3A;DNM1;SCN1A	RHOC GTPase cycle	DIAPH1
Axon guidance	TIAM1;MYH9;SCN3A;DNM1;SCN1A	Cell death signalling via NRAGE, NRIF and NADE	TIAM1
Nervous system development	TIAM1;MYH9;SCN3A;DNM1;SCN1A	Sensory processing of sound	MYH9
Developmental Biology	TIAM1;MYH9;SCN3A;DNM1;MED17;SCN1A	G alpha (12/13) signalling events	TIAM1
EPH-Ephrin signaling	TIAM1;MYH9;DNM1	Transcriptional regulation of white adipocyte differentiation	MED17
Cardiac conduction	SCN3A;SCN1A	Post NMDA receptor activation events	GRIN2D
Semaphorin interactions	MYH9	RAC2 GTPase cycle	TIAM1
Sensory perception of sweet, bitter, and umami (glutamate) taste	SCN3A;SCN1A	Protein-protein interactions at synapses	GRIN2D
Muscle contraction	SCN3A;SCN1A	Signaling by ALK fusions and activated point mutants	MYH9
Sensory perception of taste	SCN3A;SCN1A	Signaling by ALK in cancer	MYH9
EPH-ephrin mediated repulsion of cells	TIAM1;DNM1	RAC3 GTPase cycle	TIAM1
Signaling by Receptor Tyrosine Kinases	DIAPH1;WWOX;TIAM1;DNM1	Activation of NMDA receptors and postsynaptic events	GRIN2D
Activated NTRK2 signals through CDK5	TIAM1	p75 NTR receptor-mediated signalling	TIAM1
APEX1-Independent Resolution of AP Sites via the Single Nucleotide Replacement Pathway	PNKP	Innate Immune System	DIAPH1;MYH9;DNM1
Signaling by NTRKs	TIAM1;DNM1	RSV-host interactions	MED17
RHOA GTPase cycle	DIAPH1;TIAM1	Signaling by NTRK1 (TRKA)	DNM1

CD163 mediating an anti-inflammatory response	MYH9	PPARA activates gene expression	MED17
Formation of annular gap junctions	DNM1	Regulation of lipid metabolism by PPARalpha	MED17
Negative regulation of activity of TFAP2 (AP-2) family transcription factors	WWOX	MHC class II antigen presentation	DNM1
Gap junction degradation	DNM1	Membrane Trafficking	MYH9;DNM1
Activation of the TFAP2 (AP-2) family of transcription factors	WWOX	Respiratory Syncytial Virus Infection Pathway	MED17
Retrograde neurotrophin signalling	DNM1	RHO GTPases Activate Formins	DIAPH1
ERBB2 Regulates Cell Motility	DIAPH1	Clathrin-mediated endocytosis	DNM1
RHO GTPases Activate ROCKs	MYH9	Toll Like Receptor 4 (TLR4) Cascade	DNM1
RHO GTPases activate CIT	MYH9	Leishmania phagocytosis	MYH9
Sema4D induced cell migration and growth-cone collapse	MYH9	FCGR3A-mediated phagocytosis	MYH9
Sensory Perception	MYH9;SCN3A;SCN1A	Parasite infection	MYH9
Unblocking of NMDA receptors, glutamate binding and activation	GRIN2D	Regulation of actin dynamics for phagocytic cup formation	MYH9
Ras activation upon Ca2+ influx through NMDA receptor	GRIN2D	CDC42 GTPase cycle	TIAM1
Synaptic adhesion-like molecules	GRIN2D	Death Receptor Signaling	TIAM1
Negative regulation of NMDA receptor-mediated neuronal transmission	GRIN2D	Anti-inflammatory response favouring Leishmania parasite infection	MYH9
RHO GTPases activate PAKs	MYH9	Leishmania parasite growth and survival	MYH9
Signal Transduction	DIAPH1;WWOX;TIAM1;MYH9;DNM1;GRIN2D	Toll-like Receptor Cascades	DNM1
Sema4D in semaphorin signaling	MYH9	Fcgamma receptor (FCGR) dependent phagocytosis	MYH9

Signaling by Rho GTPases	DIAPH1;TIAM1;MYH9	Vesicle-mediated transport	MYH9;DNM1
Long-term potentiation	GRIN2D	RAC1 GTPase cycle	TIAM1
Signaling by Rho GTPases, Miro GTPases and RHOBTB3	DIAPH1;TIAM1;MYH9	Neurotransmitter receptors and postsynaptic signal transmission	GRIN2D
Signaling by NTRK2 (TRKB)	TIAM1	Parasitic Infection Pathways	MYH9
CREB1 phosphorylation through NMDA receptor-mediated activation of RAS signaling	GRIN2D	Leishmania infection	MYH9
EPHA-mediated growth cone collapse	MYH9	Transmission across Chemical Synapses	GRIN2D
RHO GTPase Effectors	DIAPH1;MYH9	RAF/MAP kinase cascade	GRIN2D
Nuclear signaling by ERBB4	WWOX	MAPK1/MAPK3 signaling	GRIN2D
Transcriptional regulation by the AP-2 (TFAP2) family of transcription factors	WWOX	DNA Repair	PNKP
Resolution of Abasic Sites (AP sites)	PNKP	MAPK family signaling cascades	GRIN2D
RHOF GTPase cycle	DIAPH1	Neuronal System	GRIN2D
EPHB-mediated forward signaling	TIAM1	Generic Transcription Pathway	WWOX;MED17
Assembly and cell surface presentation of NMDA receptors	GRIN2D	Immune System	DIAPH1;MYH9;DNM1
Gap junction trafficking	DNM1	Neutrophil degranulation	DIAPH1
Recycling pathway of L1	DNM1	Infectious disease	MYH9;MED17
Gap junction trafficking and regulation	DNM1	Diseases of signal transduction by growth factor receptors and second messengers	MYH9
RHOD GTPase cycle	DIAPH1	RNA Polymerase II Transcription	WWOX;MED17
Sensory processing of sound by outer hair cells of the cochlea	MYH9	Gene expression (Transcription)	WWOX;MED17
Signaling by ERBB2	DIAPH1	GPCR downstream signalling	TIAM1
NRAGE signals death through JNK	TIAM1	Signaling by GPCR	TIAM1
Neurexins and neuroligins	GRIN2D	Metabolism of lipids	MED17

RHO GTPases activate PKNs	MYH9	Adaptive Immune System	DNM1
Signaling by ERBB4	WWOX	Disease	MYH9;MED17
Sensory processing of sound by inner hair cells of the cochlea	MYH9	Viral Infection Pathways	MED17
RHOB GTPase cycle	DIAPH1	Metabolism	MED17
RHO GTPase cycle	DIAPH1;TIAM1		
